# Psychological Profile in Female Cyclists and Its Relationship with Age, Training Parameters, Sport Performance, and Injury Incidence

**DOI:** 10.3390/ijerph18073825

**Published:** 2021-04-06

**Authors:** Lucía Abenza-Cano, Linda H. Chung, Raquel Vaquero-Cristóbal, Adrián Mateo-Orcajada, Alberto Encarnación-Martínez

**Affiliations:** 1 Faculty of Sport, Catholic University San Antonio of Murcia (UCAM), Av. De los Jerónimos, 135, 30107 Murcia, Spain; labenza@ucam.edu (L.A.-C.); amateo5@alu.ucam.edu (A.M.-O.); 2 Red Española de Investigación del Rendimiento Deportivo en Ciclismo y Mujer (REDICYM) del Consejo Superior de Deportes (CSD), C. de Martín Fierro, 5, 28040 Madrid, Spain; alberto.encarnacion@uv.es; 3 Research Center for High Performance Sport, Catholic University San Antonio of Murcia (UCAM), Av. de los Jerónimos, 135, 30107 Murcia, Spain; 4 Kinanthropometry International Chair, Catholic University San Antonio of Murcia (UCAM), Av. de los Jerónimos, 135, 30107 Murcia, Spain; 5 Research Group in Sports Biomechanics (GIBD), Department of Physical Education and Sports, University of Valencia, C/Gascó Oliag, 3, 46010 Valencia, Spain

**Keywords:** competition, cycling, psychological profile, sports performance, training, women

## Abstract

Previous studies have highlighted the importance of psychology on sports performance and its relationship with the incidence of sport injuries. The objectives of the present investigation were: (1) to analyze the psychological profile of female cyclists as a function of age, training parameters, sport performance, and injuries suffered and (2) to design a model to predict their psychological profile. Sixty-one female cyclists participated in the study. Differences were found as a function of a competitive category for team cohesion (F = 5.035; *p* = 0.002), sport level effect on performance evaluation (F = 5.030; *p* = 0.004) and team cohesion (F = 64.706; *p* = 0.000), the effect of having reached the podium in the last competition on performance evaluation (t = 2.087; *p* = 0.041) and motivation (t = 4.035; *p* = 0.000), and injury severity on stress management (F = 6.204; *p* = 0.008). The factors that affected the psychological profile of the female cyclists the most, in addition to the independent psychological parameters, were the number of podiums in the last year and the years of cycling experience. In conclusion, there is an interaction between the psychological profile, sociodemographic variables, training, performance, and injuries suffered in female cyclists.

## 1. Introduction

Previous studies have highlighted the importance of psychology on sport performance [1,2]. Consequently, sports psychologists have been progressively incorporated into the sports field [2,3,4]. An increase in an athlete’s psychological performance improves stress and anxiety management as well as propagates motivation and self-confidence in competitive situations, thus positively influencing sports performance [5]. In contrast, inordinate psychological stress causes mood swings, depression, and anxiety symptoms that negatively affect the athlete’s sport performance as well as daily life [6,7].

According to previous studies, athletes show adequate levels of motivation, stress control or acceptance of external evaluation, but the margin for improvement is large [8,9]. Psychological training programs that are appropriate to the athlete’s characteristics have been implemented and have obtained favorable results in the control of competitive anxiety, emotional control, attentional control, and flow state as well as a decrease in pessimism and anxiety when confronted with sport situations [10,11,12,13]. However, not all psychological interventions are similar for all athletes, since each athlete has a different psychological profile that depends largely on the sport modality, sport category, playing position, and age [8,14,15].

Differences according to sport modality, sport category, playing position or age can occur in any of the five categories of the psychological profile: stress management, the influence of performance evaluation, motivation, mental ability, and team cohesion [16]. Stress management is the ability of the athlete to use his/her own’s psychological resources to manage the stress related to his/her participation in sport competitions. This dimension includes two categories: (1) the characteristics of the athlete’s response to the demands of sport and (2) the situations that generate stress and require control before and during sport practice. The influence of performance evaluation refers to the perception of high control over the impact of a negative evaluation on a performance by the athlete or others and includes two categories: (1) the characteristics of the athlete’s response when self-evaluating his/her performance or by people close to him/her and (2) the antecedents that can lead to an evaluation of a performance by the athlete. Motivation is defined as the athlete’s commitment to the sport as well as the daily interest in sports practice, leading to an interest in surpassing oneself daily, the establishment and achievement of goals, the cost–benefit of sports practice, and the importance of sport in relation to other life activities. Mental ability refers to coping resources or psychological skills, such as goal setting or imaginative rehearsal, that can enhance performance. Finally, team cohesion indicates the degree of integration and the feeling of belonging of the athlete to his/her team [16].

Previous research has analyzed the psychological profile of athletes in various sports modalities [9,14,17,18]. For example, male soccer players report higher levels of stress control, motivation, and team cohesion than male basketball and male rugby players, while male rugby players show a greater influence on performance evaluation and mental ability than players in other sport modalities [9]. Similarly, male triathletes score higher in attention, motivation, and attitude than male golfers and male soccer players [19]. When comparing male and female triathletes and male and female cyclists, Olmedilla [17] demonstrated that male and female triathletes have higher scores in motivation, stress control, and mental skills and have the ability to accept evaluations of their performance. With regards to the specific position occupied within the sports team, Olmedilla [14] observed in their study with male handball players that the goalkeeper had the highest scores in all psychological characteristics, thus obtaining a better psychological profile for sports performance compared to the rest of the teammates. Regarding the competitive level, both Ruiz-Esteban et al., who examined female soccer players, and Ramirez and Prieto, who investigated a sample of boys and girls from a number of sports, found that athletes that have qualities to reach the elite level but have not yet achieved it present lower values of mental ability and motivation than athletes who have already reached the elite in the same category [15,20]. Likewise, male triathlon professionals present higher scores in all psychological dimensions assessed compared to amateurs [17]. However, studies that have examined the differences in the psychological profile by age present contradictory results. Some investigations that observed a sample of boys and girls between the ages of 12 and 20 years that represent twenty-two sporting disciplines found no differences amongst the different ages of athletes with regards to the psychological profile [20]. Other studies examining female soccer players between 13 and 17 years have pointed out that some parameters, such as team cohesion in the group, is higher in younger athletes [21].

In addition to its influence on sport performance, the psychological profile is related to the risk of sport injuries and the ability to cope with injuries [18,22,23,24]. Previous injury, self-confidence, competitive trait anxiety, and negative coping measures are significant predictors of the number of injuries in young male and female athletes [18,24]. During the recovery process post-injury, athletes with a stronger psychological profile (i.e., higher levels of self-esteem and internal focus of control) show better performance in functional tests, which is a determinant for the athlete’s return to sport [22,23]. Like sports performance, psychological interventions are effective in reducing the number of injuries and days lost due to the fact of injury in male athletes [25]. The improvements obtained in stress and anxiety control, as well as increased pain tolerance, are determinants that favor recovery in male and female injured athletes [26].

Despite the importance of having coaches and sports psychologists know the athlete’s psychological profile in order to implement appropriate strategies to improve performance and prevent injuries [17,27], there is limited information available on the psychological profiles of cyclists. Spindler [7] observed positive mood states, lower influence of mental fatigue, and higher leadership ability among elite male cyclists compared to non-elite cyclists, while Olmedilla [17] found that female amateur cyclists presented a tendency to higher values in all categories of the psychological profile compared to male amateur cyclists, although there were no significant differences between sexes.

Previous research in the field of psychology with regards to cycling and triathlon is scarce [17,28]. It is important to establish the psychological profile in these sports modalities to provide empirically-based recommendations for coaches and athletes with the objective of optimizing sports performance [19]. Previous studies that have examined the psychological profile in cycling and/or triathlon [17,29] have not shown significant differences between males and females, suggesting that the psychological needs in training and competition are similar in both sexes. However, the sample sizes of both studies were small (i.e., 33 cyclists [17] and between 72 [28] and 96 triathletes [17]), and, thus, further research is needed to obtain more information on this subject [29]. In addition, the characteristics of men’s and women’s cycling competitions are very different in terms of days of competition and distances covered. For example, the men’s Tour de France consists of 21 stages with an average of 161 km/stage, while the women’s counterpart consists of a one-stage race of 96 km. This aspect will also condition the psychological characteristics that determine performance [30].

To our knowledge, there are no studies that have analyzed the psychological profile of female cyclists exclusively and its relationship with sociodemographic factors, performance, and history of sports injuries. Therefore, the objectives of the present study were: (1) to analyze the psychological profile of female cyclists as a function of age, training parameters, sport performance, and injuries suffered and (2) to design a prediction model of their psychological profile. Based on previous research carried out in other sports modalities [15,17,20,22,23], we hypothesized that older cyclists, with a higher level of performance and who have suffered sports injuries in the past, would show higher scores in the dimensions of the psychological profile.

## 2. Materials and Methods

### 2.1. Participants

The calculations for establishing the sample size were performed using RStudio 3.15.0 software (RStudio Inc., Boston, MA, USA). The significance level was set at α = 0.05. The standard deviation (SD) was established based on previous studies that examined stress control (mean SD = 16.7), influence of performance evaluation (mean SD = 9.39), motivation (mean SD = 5.55), and mental skills (mean SD = 5.49) [17]. The estimated errors (d) were 4.19 for stress control, 2.36 for the influence of performance evaluation, 1.39 for motivation, and 1.37 for mental skills. The calculated sample needed for this study was 61 subjects.

The cyclists were recruited by e-mail through the Spanish Network for Research on Sports Performance in Cycling and Women (REDICYM) of the Supreme Sports Council (CSD). Participants were volunteers from Spain, and the selection of the participants was non-probabilistic for convenience. The inclusion criteria were: (1) female; (2) had participated in national and/or international competitions; (3) federated at the regional or national level; and (4) practiced federated cycling for at least two years. A total of 71 female cyclists participated in the present study.

### 2.2. Procedure

The design of the present study was descriptive and cross-sectional with data collected at a single point in time. The Institutional Ethical Committee reviewed and authorized the protocol designed for data collection, according to the Code of the World Medical Association (number CE062002). The statements of the Declaration of Helsinki were followed during the entire process.

All the participants were informed about the procedures and those who wanted to participate in the present study signed an informed consent form before the start of the study. For underage participants, a parent or guardian was responsible for signing the informed consent form to allow participation. Afterwards, each volunteer was sent an online questionnaire created in Google Forms and were asked to fill it out in a relatively quiet environment while not under training or competition pressure and without the presence of their coach or other cyclists. The participants did not receive any additional explanation when completing the questionnaire and their submission was done anonymously. The time to complete the questionnaire ranged from 20 to 30 min.

The questionnaire consisted of two blocks. In the first block, participants completed the questionnaire of psychological characteristics related to sports performance [16]. This questionnaire is an instrument derived from the Psychological Skills Inventory for Sports (PSIS) called the “Questionnaire of Psychological Characteristics Related to Sports Performance (CPRD)” [16] and whose objective was to evaluate a series of psychological skills involved in performance in competitive sport. It consisted of 55 items distributed across the five factors identified earlier: stress control, influence of performance evaluation, motivation, mental ability, and team cohesion. The CPRD questionnaire has been shown to have a high internal consistency with Cronbach’s alpha values of 0.85 in different sports that included individual sports and females [16,31,32]. This questionnaire was chosen because of its great utility in the evaluation of the psychological characteristics involved in sport performance, continuity in competitive sport and sport success [33], and because it has been used in previous research [15,17].

In the second block, participants answered a demographics questionnaire as well as an injury questionnaire to collect relevant information regarding the frequency and severity of the injuries suffered. This demographic questionnaire asked for age, weekly training hours, weekly training frequency, years of experience, competitive category, sporting successes in the last year (i.e., the number of competitions in which they had reached the podium), if they were or had been a member of the national team and if they had achieved a podium placement in the last competitions. After this, data were collected on the sports injuries suffered in the last year, recording the number of times they had been injured in the last year and the degree of severity of each of the injuries suffered, through the previously validated questionnaire. Functional criteria were used to assess the severity of the injuries [34], differentiating between mild (interruption for at least one day of training and requirement of treatment), moderate (interruption of training and competitions between 6 and 30 days and requirement of treatment), serious (involving between one and three months of sick leave, some hospitalization and even surgery), and very serious (consisting of four months of sick leave) injuries. It was also recorded whether they had recovered from these injuries. The sports injuries questionnaire regarding sports injuries was made ad hoc following the structure of previous questionnaires on sports injuries [35,36,37]. The sports injuries questionnaire has been previously designed and validated in a pilot study, following the indications of Carretero-Dios and Pérez [38] to obtain correct validity of content, comprehension, and construct. The pilot study was conducted on cyclists and allowed for the design and validation of the questions related to sports injuries suffered. Content validity was examined by a panel of experts who assessed the adequacy of the construct, the dimensions and the qualitative and quantitative items. Items with quantitative ratings of less than seven on the Likert scale of 1 (not at all adequate, comprehensive or concrete) to 10 (extremely adequate, comprehensive and concrete) and items with qualitative ratings of less than 50% agreement were eliminated by the experts. The final questionnaire, which consisted of 5 items, obtained a concordance index of 0.92 with agreement in all items exceeding 85%. In addition, the reliability of the questions of the sports injuries questionnaire was analyzed in the pilot study, presenting Cronbach’s alpha reliability coefficients above 0.75, revealing that the instrument had an adequate internal consistency with composite reliability indexes above 0.70 and maximum shared variance (MSV) above 0.50.

After data collection, the score was calculated for each of the five dimensions collected by the CPRD in accordance with the methodology used in previous research [16]. The scores ranged between 4 and 80 for stress control, between 2 and 45 for influence of performance evaluation, between 7 and 31 for motivation, between 8 and 34 for mental ability, and between 0 and 24 for team cohesion [16]. In addition, the sports level of the cyclists was determined by the hours of weekly practice according to the classification of De Pauw [39]. These authors include five sport levels, 1 being the lowest and 5 being the highest, which established the professional level of the female cyclists. For the age groups, the competitive sports categories were determined using the same criteria as García-Naveira and Remor [40] with their soccer players, where they classified the five groups: 13–16 years (infantile and cadet), 17–18 years (juvenile), 19–22 years (under 23), 23–30 years (elite), and >30 years (master).

### 2.3. Statistical Analysis

Statistical analysis was performed using the SPSS statistical package (v.25.0; SPSS Inc., Chicago, IL, USA). The normality of the distribution was checked with the Kolmogorov–Smirnov test. The kurtosis analysis showed a platykurtic distribution for all the variables. All the variables included in the analysis followed a normal distribution, so a parametric statistics test was performed. A descriptive statistics test was performed for all of the variables, while counts and percentages were calculated for categoric variables. For the analysis of the differences in the psychological profiles of the cyclists according to age group, sport level, and severity of the injury, a one-way analysis of variance (ANOVA) of one factor was carried out, performing a Bonferroni pairwise comparison in the variables with statistical significance with an adjusted value of *p* < 0.016 (severity of the injury) and *p* < 0.010 (age group and sport level). The confidence interval (CI) of the differences (95% CI) was included. Partial eta squared was used to calculate the effect size (ES) and was defined as small for ≥0.10, moderate for ≥0.30, large for ≥1.2 or very large for ≥2.0 [41]. To determine the differences in the psychological profile of the athletes who were invited to the national team, who had reached the podium in the last competitions, and who had suffered injuries in the last year, a Student’s *t*-test for independent samples was used. Cohen’s d was calculated to determine the effect size in these cases, being small when d < 0.2, moderate when d < 0.8, and large when d > 0.8 [42]. The correlation values between the different measures were obtained using Pearson’s correlation coefficient. For the variables that reached significance in the Pearson correlation, a linear regression was performed to predict the variables that could have the greatest influence on the psychological profile of the female cyclists. The prediction equations obtained from the model follow the structure y = b_0_ + b_1x_, where “y” represents the independent variable, “x” the dependent variable, and b_0–1_ denotes the parameters that are held constant. A value of *p* < 0.05 was established to determine statistical significance.

## 3. Results

Table 1 shows the characteristics of the female cyclists with respect to age, training variables, psychological characteristics, and sports injuries.

Table 2 shows the differences in the psychological characteristics related to sport performance as a function of age group, sport level, the national selected category, having reached the podium in the last few competitions, having suffered injuries in the last year, severity of injuries suffered in the previous season, and having fully recovered from the injuries suffered. Differences between age groups with a small effect size were found for team cohesion. Subsequent Bonferroni adjustment showed that female cyclists in the 13 to 16 year old group had significantly less team cohesion than the 19 to 22 year old group (*p* = 0.007). Differences with a small effect size were also found between sport level groups for the influence of performance evaluation and team cohesion, and post-hoc tests showed that female cyclists with a sport level of 3 had significantly higher scores on the influence of performance evaluation than cyclists with a sport level of 5 (*p* = 0.005). There were differences between all pairs of sport level groups for team cohesion, with lower sport levels showing lower scores (*p* between 0.005 and 0.000). In addition, cyclists who had been on the podium in recent competitions showed a significantly higher score for the influence of performance evaluation (*p* = 0.041) and motivation (*p* = 0.000) with a moderate to large effect size compared to the group of cyclists who had not. Regarding injury severity, significant differences with a moderate effect size were found for stress control, with the Bonferroni adjustment showing that female cyclists who had sustained moderate injuries had higher scores than cyclists with mild severity (*p* = 0.007).

The correlational analysis of the psychological profile of the cyclists, the sports performance variables, and the injuries suffered are shown in Table 3. A positive correlation was found between stress control and the influence of performance evaluation and mental ability. There was also a negative correlation between motivation and age, and a positive correlation between motivation and mental ability and the number of podiums in the last year. Regarding mental ability, there was a negative correlation with the years of sport practice. Team cohesion showed a negative correlation with the number of podiums in the last year. Finally, the hours of weekly practice correlated positively with the frequency of sport practice.

Table 4 shows the results of the linear regression analysis for the psychological profile of the female cyclists. One model was found for stress control, influence of performance evaluation, team cohesion and motivation and two models for mental ability. From these results, the following prediction equations were determined: stress control = 11.208 + (1.382 × influence of performance appraisal), influence of performance appraisal = 11.208 + (1.382 × stress control), motivation = 17.313 + (1.265 × number of podiums in the last year), mental ability = 19.105 + (−0.229 × years of experience) + (0.209 × motivation), and team cohesion = 20.543 + (−0.762 × number of podiums in the last year).

## 4. Discussion

The first objective of the present investigation was to establish the psychological profile of professional female cyclists and to analyze the differences in this profile according to age, training variables, sport performance, and injuries suffered in female cyclists. Attending to Gimeno et al. [16], the results obtained show percentiles lower than 55 for all the dimensions of the psychological profile of the female cyclists participating in the study, which differs from previous studies in which professional handball [14], soccer [9,43], basketball, and rugby [9] players obtained values higher than the 60th percentile. The differences found could be due to the sex of the participants, since previous studies were conducted in male athletes. Sex differences in the psychological profile and psychological variables, such as competitive anxiety, emotional stability or emotion control, have been reported [20,44,45,46]. Previous research also shows differences between individual and group sports modalities [46]. Therefore, future research is needed to truly establish the differences in the psychological profile between male and female athletes, since the existing results are not generalizable [17] as well as between different sport modalities.

An important finding of the present research is that, in general, age does not seem to influence the psychological profile of female cyclists. These results are similar to those found in previous research conducted in judo and handball [20,44,47]. This could be due to the fact that the age groups established for the analysis include disparate groupings, with the infantile, cadet, and juvenile categories being three years in duration, while the two senior categories are of longer duration. The research by Cazorla [48] in triathletes also showed this limitation, so further studies that include more homogeneous age bands are necessary. However, female cyclists in the infantile and cadet categories showed significantly less group cohesion than the U-23 group with no differences between the rest of the groups. Reche [49] found similar results in other psychological variables when analyzing a group of nationally ranked fencers with the most experienced athletes obtaining higher scores in attention and control of emotions. In the present study, only differences in group cohesion according to age were found, and this could be due to the predominant reasons for practicing in each of the age groups. For example, while adult women practice for fun and social relationship [50], young athletes do so with a more marked competitive goal [51,52], producing in this latter group a lower concern for team performance in favor of individual benefit.

Another relevant finding was that women cyclists with lower levels, determined based on weekly practice hours following the methodology of De Pauw [39], showed higher scores in the influence of performance evaluation, although significant differences were only found between groups 3 and 5. The influence of performance evaluation is a dimension of the psychological profile that can produce modifications in other psychological variables of athletes, generating a positive or negative influence on performance depending on personal characteristics [53]. Thus, it may be that female cyclists with a lower sporting level and with lower expectations of success are less influenced by performance evaluation because they do not pursue a performance goal, so the result obtained in competitions will not negatively affect these athletes compared to those who opt to obtain sporting success during the season. Similar results were obtained by Marsillas et al. [54] who, despite not establishing significant differences, found a higher score in the evaluation of performance in medium/high-level athletes compared to elite athletes in different sports modalities. On the other hand, the higher the level of the cyclists, the higher the group cohesion score. This may be because a higher sport level requires better functioning as a team in pursuit of a common goal, and this is supported by Marcos et al. [55], as they established that basketball players who perceived greater team cohesion presented higher expectations of success and greater sports performance.

It should be noted that significant differences were found in the psychological profiles according to the recent sporting success of female cyclists, evaluated via reaching the podium in the last competitions. Female athletes who had reached the podium in recent competitions obtained significantly higher values in the influence of performance evaluation and motivation. This could be due to several reasons. On the one hand, these results could indicate that athletes with greater mental strength and stronger psychological profiles obtain better sports results. Previous studies that have carried out psychological interventions have obtained favorable results in the control of competitive anxiety, emotional and attentional control, as well as in the decrease of pessimism and anxiety, and in the management of the influence of performance evaluation and stress control, which could help in improving sports performance [10,11,12,56,57,58]. However, it could also be that the achievement of success leads to a modification of the athlete’s psychological profile, more specifically to higher values in the influence of performance evaluation and motivation. In this regard, the results of previous research are not conclusive, as Sánchez [31] found that female gymnasts who had achieved success during their sports career had higher scores in the influence of performance evaluation which would indicate that these athletes progressively acquire more capacity to analyze their performance and work. However, González-García and Pelegrín [59] found no significant differences in any of the dimensions of the psychological profile of table tennis players who had achieved international success when compared to those who had not. Therefore, further studies are needed to determine the existence of differences in the psychological profile of successful and unsuccessful players, depending on the sport modality practiced.

Regarding the injuries suffered, the results of the present investigation showed that those women cyclists who had suffered moderate injuries showed significantly greater stress control than those who had suffered minor injuries. One possible explanation for this fact is that self-confidence, included within stress control, can produce a decrease in the perception of risk, resulting in a higher incidence of injury due to the overexertion and risk behaviors carried out [60,61]. Therefore, self-confidence can induce risk behaviors, increasing the possibility of suffering a serious injury, but it can also generate attitudes of control of negative emotions, which would be related to lower risk behaviors, decreasing vulnerability to injury [62]. These findings raise the possibility that the type of sport and level of competition are determinants when considering the psychological profile as a function of sports injuries, since previous studies [63] have found differences in the psychological profile of handball players according to the type and severity of the injury, where athletes with higher levels of motivation and mental ability suffered more moderate injuries. In addition, all of the variables of the athlete’s environment that could influence the injury process should be considered, since Andreu [64] found that athletes with a profile of vulnerability to injury suffered fewer and less severe injuries than those who did not have this profile, which would imply that the psychological profile is not the only determining factor in injuries.

Based on the results obtained, the study hypothesis can be partially accepted, since older athletes and sports performance showed greater team cohesion but lower scores in the influence of performance evaluation. No significant differences were observed in the dimensions of the psychological profile of cyclists who have suffered injuries in the last year.

The second objective of the present research was to determine the prediction equations of the psychological profile of female cyclists. The linear regression model makes it possible to determine the degree of dependence between the dependent and independent variables (i.e., establishing the impact of changes in the dependent variables on the independent variable). The practical importance of the linear regression models is due to the fact that the equations makes it possible to calculate the value of an independent variable by knowing the value of the dependent variable that is able to explain it, since the parameters are held constant [41].

It was found that stress control depended primarily on the assessment of sport performance and that the assessment of sport performance depended primarily on stress control, there being a direct relationship between the two factors. Previous studies found similar results to those of the present study, where athletes who had higher stress control scores being those who were able to cope more positively with performance evaluation performed by themselves or by their environment [65]. This is relevant when considering that the bidirectional relationship between both variables has an influence on sport performance as indicated by Pacheco and Gómez [66] in a study with elite soccer players in which athletes who had greater control over negative evaluations showed superior sport performance.

On the other hand, the variable that most explained the motivation score was the number of podiums in the last season, where a direct relationship was observed between these factors. Attending to the achievement goals theory [67] and considering the competitive environment in which the present research is framed, the relationship between motivation and the number of podiums could be explained because women cyclists are result-oriented, trying to achieve success in each competition. Previous research conducted in taekwondo [68] and soccer [40] has shown this relationship, where athletes with a higher motivational level were those who obtained more sporting success.

Regarding the factors that conditioned mental ability, there was a direct relationship between mental ability and motivation, while there was an indirect relationship with years of experience. Lavarello [65] corroborates this finding where they observed a slight, positive relationship between mental ability and motivation. If mental ability is considered as the ability to use psychological skills that can favor performance [16], it is logical that there is a relationship with motivation because Mouratidis and Michou [69] determined that athletes with higher autonomous motivation had better coping of sport situations, so they were able to optimally use their psychological skills. Regarding years of experience, Godoy-Izquierdo [70] found that athletes with more years of sport experience reported lower scores on numerous psychological skills compared to those with shorter sport trajectories. This could be due to the fact that more experienced athletes are the ones who present higher levels of emotional exhaustion and reduced personal fulfillment, both dimensions belonging to burnout, which was demonstrated in women’s field hockey [13]. Benedicto and Garcés de los Fayos [71] observed that the mental ability of athletes with burnout was significantly lower than that of athletes who did not have this syndrome, so there could be an indirect relationship between mental ability and years of experience.

Finally, an indirect relationship was found between the number of podiums and team cohesion. This may be explained by the fact that individual performance of the athletes was considered and not group performance. Athletes who participate in competition do so by trying to achieve personal sports performance goals during practice which would allow them to achieve the proposed objectives, without these being related to a common performance objective, reducing the importance of team cohesion when the individual performance of each athlete is evaluated [72].

There were limitations to the present study. Although the sample size was much higher than previous research [58,73,74], it would be interesting to increase the sample in future research, especially to perform the analyses based on categorical variables. Although the sample size was larger compared to previous research, it was still too small to generalize the results to the population of women cyclists; thus, the results should be treated with caution. On the other hand, the results obtained in the present investigation are a first approximation to the psychological variables that can be determinant and that can establish significant differences in the sport performance of the female athletes that practice cycling. However, since this study was carried out using a cross-sectional observational methodology, it would be necessary to carry out psychological intervention programs to obtain information on their effect on the psychological profile of women cyclists.

## 5. Conclusions

There are differences in the psychological characteristics related to the sports performance of women cyclists according to age, competitive level, having been successful in recent competitions, and the severity of injuries suffered in the last year. These findings provide evidence on the factors that may influence the psychological profile of female cyclists, which can, in turn, have a great impact on sports performance. In addition, the psychological profile obtained in this study may be useful for sports psychologists in this field to work adequately with female athletes.

## Figures and Tables

**Table 1:** Characteristics of women cyclists.

	Variable	Results
Age	Age (mean ± SD)	23.95 ± 9.29
	Age distribution by categories (*n* and %)	
	Infantile and cadet (13–16 years)	19 (31.1%)
	Juvenile (17–18 years)	10 (16.4%)
	Under 23 (19–22 years)	7 (11.5%)
	Elite (23–30 years)	8 (13.1%)
	Master (>30 years)	17 (27.9%)
Training and performance variables	Weekly practice hours (mean ± SD)	11.97 ± 4.41
	Frequency of weekly practice (mean ± SD)	2.66 ± 0.63
	Years of sports practice (mean ± SD)	7.85 ± 4.19
	Number of podiums in the last year (mean ± SD)	1.40 ± 1.28
	Sports level (*n* and %)	
	1	4 (6.6%)
	2	0 (0.0%)
	3	11 (18.0%)
	4	6 (9.8%)
	5	40 (65.6%)
	Podiums in last competitions (*n* and %)	
	Yes	40 (65.6%)
	No	21 (34.4%)
	Called up with the Spanish national team (*n* and %)	
	Yes	12 (19.7%)
	No	49 (80.3%)
Psychological characteristics related to sports performance	Stress management (mean ± SD)	44.28 ± 13.62
	Influence of performance evaluation (Mean ± SD)	23.93 ± 7.51
	Motivation (mean ± SD)	19.05 ± 4.12
	Mental ability (mean ± SD)	21.30 ± 3.22
	Team cohesion (mean ± SD)	19.39 ± 2.88
Sports injuries	Sports injuries in the last year (*n* and %)	
	Yes	22 (36.1%)
	No	39 (63.9%)
	Number of injuries in the last year (mean ± SD)	1.50 ± 0.67
	Severity of injury (*n* and %)	
	Slight	8 (36.4%)
	Moderate	9 (40.9%)
	Serious	5 (22.7%)
	Cyclists fully recovered from last season’s injuries (*n* and %)	
	Yes	18 (66.7%)
	No	9 (33.3%)

**Table 2:** Differences in the psychological profile of female cyclists according to sport modality, age, sport level, and injury severity (mean ± standard deviation).

	Stress Management	Influence on Performance Evaluation	Motivation	Mental Ability	Team Cohesion
**Age**					
**13–16**	44.93± 11.18	25.07 ± 5.17	19.67 ± 1.70	21.52 ± 1.28	18.79 ± 0.63
**17–18**	42.57 ± 9.19	22.04 ± 4.41	19.00 ± 1.90	20.26 ± 1.07	19.40 ± 0.29
**19–22**	39.53 ± 11.63	20.21 ± 4.90	18.10 ± 1.79	20.61 ± 1.02	19.81 ± 0.58 **
**23–30**	44.21 ± 10.93	21.50 ± 8.87	18.49 ± 2.66	20.80 ± 1.87	19.56 ± 0.69
**>30**	47.80 ± 8.16	26.81 ± 5.62	18.95 ± 1.98	20.98± 1.19	18.93 ± 0.78
**F, *p***	0.976; *p* = 0.428	2.605; *p* = 0.045	1.047; *p* = 0.392	1.788; *p* = 0.144	5.035; *p* = 0.002
**Effect size**	0.090	0.065	0.191	0.079	0.074
**Sports level**					
**1**	51.11 ± 14.29	28.05 ± 5.20	20.17 ± 2.30	21.22 ± 1.62	17.63± 0.08 **
**2**					
**3**	48.63 ± 7.64	29.10 ± 3.62	19.10 ± 1.93	21.40 ± 1.38	18.33 ± 0.18 **
**4**	40.68 ± 7.97	22.12 ± 6.55	19.39 ± 1.65	20.90 ± 1.60	18.82± 0.00 **
**5**	43.47 ± 10.28	22.52 ± 5.80 **	18.82 ± 2.01	20.83 ± 1.26	19.57 ± 0.42
**F, *p***	1.655; *p* = 0.187	5.030; *p* = 0.004	0.646; *p* = 0.589	0.571; *p* = 0.637	64.706; *p* = 0.000
**Effect size**	0.091	0.080	0.017	0.074	0.062
**Have you been called up to the Spanish national team?**					
**Yes**	45.08 ± 13.18	22.83 ± 6.65	19.25 ± 4.77	21.25 ± 3.70	18.67 ± 3.31
**No**	44.08 ± 13.85	24.20 ± 7.75	19.00 ± 4.00	21.31± 3.13	19.57 ± 2.77
**t, *p***	0.227; *p* = 0.822	−0.563; *p* = 0.575	0.187; *p* = 0.852	−0.054; *p* = 0.957	−0.976; *p* = 0.333
**d**	0.07	0.19	0.06	0.02	0.29
**Have you been on the podium in recent competitions?**					
**Yes**	46.45 ± 13.48	25.35 ± 7.38	20.43 ± 4.07	21.70 ± 3.63	18.95± 3.03
**No**	40.14 ± 13.22	21.24 ± 7.16 *	16.43 ± 2.75 **	20.52 ± 2.09	20.24 ± 2.41
**t, *p***	1.748; *p* = 0.086	2.087; *p* = 0.041	4.035; *p* = 0.000	1.367; *p* = 0.177	−1.687; *p* = 0.097
**d**	0.47	0,.56	1.15	0.40	0.47
**Have you suffered any injuries in the last year?**					
**Yes**	48.14 ± 12.94	25.73 ± 7.97	18.59 ± 3.62	21.77 ± 3.01	19.41 ± 2.70
**No**	42.10 ± 13.67	22.92 ± 7.15	19.31 ± 4.40	21.03 ± 3.34	19.38 ± 3.01
**t, *p***	1.687; *p* = 0.097	1.412; *p* = 0.163	−0.650; *p* = 0.518	0.869; *p* = 0.388	0.032; *p* = 0.975
**d**	0.45	0.37	0.18	0.23	0.01
**Injury severity**					
**Slight**	39.51 ± 9.19 **	23.09 ± 4.57	18.60 ± 1.60	20.77 ± 0.89	18.90 ± 0.78
**Moderate**	54.43 ± 8.31	28.86 ± 5.13	19.69 ± 2.13	21.37 ± 1.35	18.91 ± 0.84
**Serious**	45.79 ± 8.89	26.16 ± 5.54	18.81 ± 2.30	21.15 ± 1.09	18.92 ± 0.50
**F, *p***	6.204; *p* = 0.008	2.793; *p* = 0.086	0.700; *p* = 0.509	0.596; *p* = 0.561	0.001; *p* = 0.999
**Effect size**	0.233	0.395	0.020	0.019	0.187
**Have you fully recovered from the injury?**					
**Yes**	46.94 ± 12.59	25.17 ± 8.14	18.33 ± 3.71	21.72 ± 3.14	19.78 ± 1.83
**No**	45.33 ± 14.55	24.56 ± 6.78	18.33 ± 4.00	20.67 ± 2.55	19.11 ± 3.59
**t, *p***	0.298; *p* = 0.768	0.194; *p* = 0.848	0.000; *p* = 1.000	0.872; *p* = 0.391	0.645; *p* = 0.524
**d**	0.12	0.08	0.01	0.37	0.24

* *p* < 0.05; ** *p* < 0.01.

**Table 3:** Correlational analysis of the study variables.

	Influence on Performance Evaluation	Motivation	Mental Ability	Team Cohesion	Age	Practice Hours	Frequency of Sports Practice	Years of Sports Practice	Number of Podiums in the Last Year	Number of Injuries in the Last Year
Stress management	0.762 **	0.173	0.305 *	−0.184	0.183	−0.116	−0.133	0.010	0.212	0.085
Influence of performance evaluation		0.024	0.248	−0.168	0.188	−0.248	−0.252	−0.115	0.194	−0.187
Motivation			0.331 **	−0.037	−0.301 *	0.030	0.071	−0.211	0.398 **	−0.186
Mental ability				0.086	0.056	−0.173	−0.097	−0.355 **	0.236	−0.294
Team cohesion					0.097	0.214	0.104	0.175	−0.369 **	−0.118
Age						−0.084	−0.222	0.079	−0.155	−0.045
Practice hours							0.711 **	0.090	0.055	0.097
Frequency of sports practice								0.069	0.043	0.211
Years of sports practice									−0.009	0.015
Number of podiums in the last year										−0.136

* *p* < 0.05; ** *p* < 0.01.

**Table 4:** Linear regression analysis for the psychological profile of female cyclists based on psychological, training, performance, and injury history parameters in female cyclists.

Analysis	*R* ^2^	*p*	Independent Variables Included	Standardized Coefficients (β)	*p*
**Stress Management**					
Model 1	0.581	0.000	Influence of performance evaluation	0.762	0.000
**Influence of Performance Evaluation**					
Model 1	0.581	0.000	Stress management	0.762	0.000
**Motivation**					
Model 1	0.159	0.002	Number of podiums in the last year	0.398	0.002
**Mental Ability**					
Model 1	0.126	0.005	Years of experience	−0.355	0.005
Model 2	0.167	0.002	Years of experience	−0.299	0.016
			Motivation	0.268	0.030
**Team Cohesion**					
Model 1	0.136	0.005	Number of podiums in the last year	−0.369	0.005

## Data Availability

The data sets generated during and/or analyzed during the current study are available from the corresponding author on reasonable request.

## References

[1] Chen X., Qiu N., Chen C., Wang D., Zhang G., Zhai L. (2020). Self-Efficacy and Depression in Boxers: A Mediation Model. Front. Psychiatry.

[2] Fuentes C.A., López-Gullón J.M., Belmonte M.J.B., Ferri J.M.V., Berenguí R., Angosto Sánchez S. (2020). Psychological dimension in the formation process of the spanish olympic wrestler. An. Psicol..

[3] Villalonga Melis T., Garcia Mas A., de las Heras Coll R., Buceta Coronado C., Smith R. (2015). Instauración y tareas de un servicio de psicología del deporte en un club de fútbol profesional. Rev. Psicol. Deport..

[4] Robles Rodríguez A., Abad Robles M.T., Robles Rodríguez J., Giménez Fuentes-Guerra F.J. (2019). Factors that affect the sport development of olympic judokas. Rev. Int. Med. Ciencias Act. Fis. Deport..

[5] Campos G.G., Valdivia-Moral P., Zagalaz J.C., Ortega F.Z., Romero-Ramos O. (2017). Influence of stress control in the sports performance: Self-confidence, anxiety and concentration in athletes. Retos.

[6] Li Y., Huang W., Zhang X., Qian F. (2018). The Outstanding Effect of Kayak Athletes Psychological Stress Relaxation from Workout for Water Sports Center in Jiangxi Province. Adv. Soc. Sci. Educ. Humanit. Res..

[7] Spindler D.J., Allen M.S., Vella S.A., Swann C. (2018). The psychology of elite cycling: A systematic review. J. Sports Sci..

[8] Álvarez-Kurogi L., Onetti W., Fernández-García J.C., Castillo-Rodríguez A. (2019). Does the psychological profile influence the position of promising young futsal players?. PLoS ONE.

[9] Olmedilla A., García-Mas A., Ortega Y.E. (2017). Psychological Characteristics for Sport Performance in Young Players of Football, Rugby, and Basketball. Acción Psicológica.

[10] Mehrsafar A.H., Strahler J., Gazerani P., Khabiri M., Sánchez J.C.J., Moosakhani A., Zadeh A.M. (2019). The effects of mindfulness training on competition-induced anxiety and salivary stress markers in elite Wushu athletes: A pilot study. Physiol. Behav..

[11] Röthlin P., Horvath S., Trösch S., Holtforth M.G., Birrer D. (2020). Differential and shared effects of psychological skills training and mindfulness training on performance-relevant psychological factors in sport: A randomized controlled trial. BMC Psychol..

[12] Scott-Hamilton J., Schutte N.S., Moyle G.M., Brown R.F. (2016). The relationships between mindfulness, sport anxiety, pessimistic attributions and flow in competitive cyclists. Int. J. Sport Psychol..

[13] Vallarino T., Reche García C. (2016). Burnout, resiliencia y optimismo en el hockey sobre hierba femenino. Cuad. Psicol. Deport..

[14] Olmedilla A., Ortega E., Garcés De Los Fayos E., Abenza L., Blas A., Laguna M. (2015). Perfil psicológico de los jugadores profesionales de balonmano y diferencias entre puestos específicos. Rev. Latinoam. Psicol..

[15] Ruiz-Esteban C., Olmedilla A., Méndez I., Tobal J.J. (2020). Female soccer players’ psychological profile: Differences between professional and amateur players. Int. J. Environ. Res. Public Health.

[16] Gimeno F., Buceta J.M., Pérez-Llanta M.D.C. (2001). El cuestionario «Características Psicológicas Relacionadas con el Rendimiento Deportivo» (CPRD): Características psicométricas. Análise Psicológica.

[17] Olmedilla A., Torres-Luque G., García-Mas A., Rubio V.J., Ducoing E., Ortega E. (2018). Psychological profiling of triathlon and road cycling athletes. Front. Psychol..

[18] González-Reyes A.A., Moo Estrella J., Olmedilla A. (2017). Características psicológicas que influyen en las lesiones deportivas de triatletas amateurs de Yucatán, México. Rev. Psicol. Deport..

[19] Reigal R.E., Delgado-Giralt J.E., Raimundi M.J., Hernández-Mendo A. (2018). Perfil psicológico deportivo en una muestra de triatletas amateurs y diferencias con otros deportes. Cuad. Psicol. Deport..

[20] Ramírez A., Prieto J.M. (2020). Análisis de las habilidades psicológicas en los deportistas promesas y talentos guipuzcoanos. Retos.

[21] Olmedilla A., Ruiz-Barquín R., Ponseti F.J., Robles-Palazón F.J., García-Mas A. (2019). Competitive psychological disposition and perception of performance in young female soccer players. Front. Psychol..

[22] Beischer S., Hamrin Senorski E., Thomeé C., Samuelsson K., Thomeé R. (2019). How Is Psychological Outcome Related to Knee Function and Return to Sport Among Adolescent Athletes After Anterior Cruciate Ligament Reconstruction?. Am. J. Sports Med..

[23] Christino M.A., Fleming B.C., Machan J.T., Shalvoy R.M. (2016). Psychological Factors Associated With Anterior Cruciate Ligament Reconstruction Recovery. Orthop. J. Sport. Med..

[24] Berengüí-Gil R., Puga J.L. (2015). Predictores psicológicos de lesión en jóvenes deportistas / Psychological Predictors of Injury in Young Athletes. Rev. Costarric. Psicol..

[25] Naderi A., Shaabani F., Gharayagh Zandi H., Calmeiro L., Brewer B.W. (2020). The Effects of a Mindfulness-Based Program on the Incidence of Injuries in Young Male Soccer Players. J. Sport Exerc. Psychol..

[26] Mohammed W.A., Pappous A., Sharma D. (2018). Effect of Mindfulness Based Stress Reduction (MBSR) in increasing pain tolerance and improving the mental health of injured athletes. Front. Psychol..

[27] Van Wijk C., Fourie M. (2017). Using psychological markers of sport injuries for navy diving training. Int. J. Sport Exerc. Psychol..

[28] Reigal R.E., Delgado-Giralt J.E., Raimundi M.J., Hernández-Mendo A. (2018). Sports psychological profile in a sample of amateur triathletes and differences with other sports. Cuad. Psicol. Deport..

[29] Hidalgo L., Tamayo I.M., Chirosa L.J. (2015). Análisis de las caracterísicas psicológicas y la toma de decisiones en un grupo de nadadores. Rev. Iberoam. Psicol. Ejerc. Deport..

[30] Union Cycliste Internationale UCI World Tour. https://www.uci.org/road/events/uci-worldtour.

[31] Jaenes Sánchez J., Carmona Márquez J., Lopa Peralto E. (2010). Evaluación y análisis de habilidades psicológicas relacionadas con el rendimiento deportivo en gimnastas de rítmica. Rev. Iberoam. Psicol. Ejerc. Deport..

[32] Ramos Cabal H., Salguero del Valle A., González Diñeiro Á., Molinero González O., Márquez S. (2018). Adaptación Para Deportes de Montaña (CPRD-M) del Cuestionario “Características Psicológicas Relacionadas con el Rendimiento Deportivo” (CPRD). Rev. Iberoam. de Diagnostico y Evaluacion.

[33] Gimeno F., Buceta J.M., Pérez-Llantada M.C. (2007). Influencia de las variables psicológicas en el deporte de competición: Evaluación mediante el cuestionario Características psicológicas relacionadas con el rendimiento deportivo. Psicothema.

[34] Díaz Pereira M.D.P. (2001). Estrés y Prevención de Lesiones.

[35] Abenza L., Olmedilla A., Ortega E., Esparza F. (2009). Lesiones y factores psicológicos en futbolistas juveniles. Arch. Med. del Deport..

[36] Olmedilla A. (2005). Factores Psicológicos y Lesiones en Futbolistas: Un Estudio Correlacional. Monografía de la Actividad Física y del Deporte.

[37] Ortín Montero F.J. (2009). Factores Psicológicos y Socio-Deportivos y Lesiones en Jugadores de Fútbol Semiprofesionales y Profesionales.

[38] Carretero-Dios H., Pérez C. (2005). Normas para el desarrollo y revisión de estudios instrumentales. Int. J. Clin. Heal. Psychol..

[39] De Pauw K., Roelands B., Cheung S.S., De Geus B., Rietjens G., Meeusen R. (2013). Guidelines to classify subject groups in sport-science research. Int. J. Sports Physiol. Perform..

[40] García-Naveira A., Remor E. (2011). Motivación de logro, indicadores de competitividad y rendimiento en un equipo de jugadores de fútbol de competición varones entre 14 y 24 años. Univ. Psychol..

[41] Hopkins W.G., Marshall S.W., Batterham A.M., Hanin J. (2009). Progressive statistics for studies in sports medicine and exercise science. Med. Sci. Sports Exerc..

[42] Cohen J. (2013). Statistical Power Analysis for the Behavioral Sciences.

[43] Olmedilla A., Ortega E., Andreu M.D., Ortín F.J. (2010). A psychological intervention programme for footballers: An evaluation of psychological skills through the CPRD. Rev. Psicol. Deport..

[44] Ruiz Barquín R. (2005). Análisis de las diferencias de personalidad en el deporte del judo a nivel competitivo en función de la variable sexo y categoría de edad deportiva. Cuad. Psicol. Deport..

[45] Ponseti Verdaguer F.J., García Más A., Cantallops Ramón J., Vidal Conti J. (2016). Gender differences in relation to anxiety associated with sports competitions. Retos.

[46] Arias I.A., Cardozo T., Aguirre-Loaiza H.H., Arenas J. (2016). Características psicológicas de rendimiento deportivo en deportes de conjunto: Diferencias entre modalidad y género. Psicogente.

[47] Martínez-Moreno A. (2017). Calidad del perfil motivacional en jugadores de balonmano. E-balonmano.com Rev. Cienc. Deport..

[48] López Cazorla R., Hernández Mendo A., Reigal Garrido R.E., Morales Sánchez V. (2015). Relaciones entre el autoconcepto y el perfil psicológico deportivo en triatletas. Cuad. Psicol. Deport..

[49] Reche García C., Cepero M., Rojas F.J. (2010). Efecto de la experiencia deportiva en las habilidades psicológicas de esgrimistas del ranking nacional español. Cuad. Psicol. Deport..

[50] Quesada D., Gómez López M. (2017). Perfiles motivacionales de los usuarios de un centro deportivo público. J. Sport Heal. Res..

[51] Boccia G., Cardinale M., Brustio P.R. (2020). Performance progression of elite jumpers: Early performances do not predict later success. Scand. J. Med. Sci. Sport..

[52] Knechtle B., Nikolaidis P.T. (2018). Sex- and age-related differences in half-marathon performance and competitiveness in the world’s largest half-marathon—The GöteborgsVarvet. Res. Sports Med..

[53] González Campos G., Campos Mesa M., Romero Granados S. (2014). Análisis de la influencia de la evaluación del rendimiento en jugadores de un equipo de fútbol. Retos Nuevas Tend. Educ. Física Deport. Recreación.

[54] Marsillas S., Ria A., Isorna M., Alonso D. (2014). Niveles de rendimiento y factores psicológicos en deportistas en formación. Reflexiones para entender la exigencia psicológica del Alto Rendimiento. Rev. Iberoam. Psicol. Ejerc. Deport..

[55] Marcos F.M.L., Calvo T.G., González I.P., Miguel P.A.S., Oliva D.S. (2010). Interacción de la cohesión en la eficacia percibida, las expectativas de éxito y el rendimiento en equipos de baloncesto. Rev. Psicol. del Deport..

[56] Tutte V., Reche C., Álvarez V. (2020). Evaluación e intervención psicológica en jugadoras de hockey sobre hierba femenino. / Evaluation and psychological intervention in female grass hockey players. Cuad. Psicol. Deport..

[57] Olmedilla A., Sánchez-Aldeguer M.F., Almansa C.M., Gómez-Espejo V., Ortega E. (2018). Entrenamiento psicológico y mejora de aspectos psicológicos relevantes para el rendimiento deportivo en jugadoras de fútbol. Rev. Psicol. Apl. Deport. Ejerc. Físico.

[58] Olmedilla A., Moreno-Fernández I.M., Gómez-Espejo V., Robles-Palazón F.J., Verdú I., Ortega E. (2019). Psychological Intervention Program to Control Stress in Youth Soccer Players. Front. Psychol..

[59] González-García H., Pelegrín A., Carballo J.L. (2017). Link between locus of control, anger and sport performance in table tennis players. Cult. Cienc. Deport..

[60] Llewellyn D.J., Sanchez X. (2008). Individual differences and risk taking in rock climbing. Psychol. Sport Exerc..

[61] Slanger E., Rudestam K.E. (1997). Motivation and disinhibition in high risk sports: Sensation seeking and self-efficacy. J. Res. Pers..

[62] Rubio V.J., Pujals C., De La Vega R., Aguado D., Hernández J.M. (2014). Autoeficacia y lesiones deportivas: Factor protector o de riesgo?. Rev. Psicol. Deport..

[63] Olmedilla A., Laguna M., Redondo A. (2011). Lesiones y características psicológicas en jugadores de balonmano. Rev. Andaluza Med. Deport..

[64] Andreu J.M.P., Ortega E., De Los Fayos E.J.G., Olmedilla A. (2014). Perfiles de personalidad relacionados con la vulnerabilidad del deportista a lesionarse. Rev. Psicol. Deport..

[65] Lavarello J. (2004). Estudio correlacional entre las variables medidas por el CPRD en futbolistas de las divisiones menores del fútbol chileno. Rev. Cuba. Med. Deport. Cult. Física.

[66] Pacheco M.P., Gómez J. (2005). Caracteristicas psicológicas y rendimiento deportivo. un estudio en jugadores bolivianos. Ajayu.

[67] Nicholls J.G. (1989). The competitive ethos and democratic education. Choice Rev. Online.

[68] Álvarez O., Estevan I., Falcó C., Hernández-mendo A., Castillo I. (2014). Perfil de habilidades psicologicas en el taekwondo. Cuad. Psicol. Deport..

[69] Mouratidis A., Michou A. (2011). Perfectionism, self-determined motivation, and coping among adolescent athletes. Psychol. Sport Exerc..

[70] Godoy-Izquierdo D., Vélez M., Pradas F. (2009). Nivel de dominio de las habilidades psicológicas en jóvenes jugadores de tenis de mesa, badminton y fútbol. Rev. Psicol. Deport..

[71] Vives Benedicto L., Garcés de los Fayos Ruiz E., Garcés de Los Fayos Ruiz E. (2004). Incidencia del síndrome de burnout en el perfil-cognitivo en jóvenes deportistas de alto rendimiento. Cuad. Psicol. Deport..

[72] Bohórquez Gómez-Millán M.R., Delgado Vega P., Fernández Gavira J. (2016). Rendimientos deportivos auto y heteropercibidos y cohesión grupal: Un estudio exploratorio. Retos.

[73] Gomez-Marcos G., Sanchez-Sanchez M. (2019). Descripción y diferencias en las variables psicológicas relacionadas con el rendimiento deportivo de triatletas y para-triatletas. Retos.

[74] Lorenzo J., Gómez M.A., Pujais C., Lorenzo A. (2012). Análisis de los efectos de un programa de intervención psicológica en jóvenes jugadores de baloncesto. Rev. Psicol. Deport..

